# Bioinformatics Study Revealed Significance of Exosome Transcriptome in Hepatocellular Carcinoma Diagnosis

**DOI:** 10.3389/fcell.2022.813701

**Published:** 2022-04-27

**Authors:** Zeng-Hong Wu, Cheng Li, You-Jing Zhang, Rong Lin

**Affiliations:** ^1^ Division of Gastroenterology, Union Hospital, Tongji Medical College, Huazhong University of Science and Technology, Wuhan, China; ^2^ Department of Otolaryngology Head and Neck Surgery, The Central Hospital of Wuhan, Tongji Medical College Huazhong, University of Science and Technology, Wuhan, China; ^3^ State Key Laboratory of Cardiovascular Disease, Department of Epidemiology, Fuwai Hospital, National Center for Cardiovascular Diseases, Chinese Academy of Medical Sciences and Peking Union Medical College, Beijing, China

**Keywords:** hepatocellular carcinoma, exosomes, data mining, exoRBase, circRNA

## Abstract

**Background:** Hepatocellular carcinoma (HCC) is one of the fifty most common cancers globally, having a high mortality rate being the second most common cause of cancer-related deaths. However, little attention has been paid to the involvement of exosomes and ceRNA in HCC.

**Method:** The study aimed to explore exosome data from exoRBase database and a free online database to estimate possible binding miRNA from mRNA, lncRNA, and circRNA and discover useful exosome biomarkers for HCC therapy.

**Results:** The results indicated that a total of 159 mRNAs, 60 lncRNAs, and 13 circRNAs were differentially expressed, with *HIST2H3C* exhibiting the highest log_2_FC change, CTD-2031P19 exhibiting the most relevant lncRNA, and CTD-2031P19 exhibiting the most relevant lncRNA. *MARCH8*, *SH3PXD2A*, has-circ-0014088, hsa-miR-186-5p, and hsa-miR-613 were identified as hub biomarkers used by Cytoscape. According to the KEGG pathway analysis results, the differentially expressed proteins were primarily enriched in the MAPK signaling network, central carbon metabolism in cancer, the glucagon signaling pathway, glutamatergic synapse, and spliceosome. Furthermore, immunohistochemical images from the Human Protein Atlas (HPA) online tool were used to directly evaluate the protein expression of *SMARCA5*, *CDC42*, and *UBC* between normal and cancer tissues, and the results showed that these three gene expressions were significantly higher in tumor tissues.

**Conclusion:** This study discovered atypical signature exosomes for HCC prognostic prediction based on an online database. The signals could mimic exosome microenvironmental disorders providing potential biomarkers for exosome treatment.

## Background

Hepatocellular carcinoma (HCC) is one of the fifty most common cancers globally, having a high mortality rate being the second most common cause of cancer-related deaths, and is diagnosed at a rate of 500,000 patients each year globally (Torre et al., 2015). The most prevalent causes of cirrhosis are viral hepatitis and nonalcoholic steatohepatitis, with roughly 80% of cases progressing to HCC (Tang et al., 2016; Coskun, 2017). Due to the recurrence of HCC, the prognosis of HCC remains bleak, with a 5-year overall survival rate of approximately 34–50% (Lang et al., 2007). Despite significant advancements in medical technology, there are no useful curative therapies for HCC patients (Jiao et al., 2018). Radical treatment can enhance the survival probability of individuals diagnosed with HCC early on and offer possible long-term treatments (De Lope et al., 2012). Various serum biomarkers such as alpha-fetoprotein (AFP) and alkaline phosphatase (AKP or ALP) are well known in clinical practice, but they have the demerit of low sensitivity and specificity (Shen et al., 2017). As a result, developing efficient biomarkers for HCC diagnosis and treatment is critical.

Exosomes are small membrane vesicles rich in lipids, proteins, RNA, and DNA secreted by most cell types due to the fusion of multivesicular late lysosomes/endosomes with the plasma membrane, thereby influencing the biological behavior of nearby or distant cells (Denzer et al., 2000). Exosomes are involved in the initiation and progression of cancer. Many studies reported that the cancer cells release more exosomes than normal cells, affecting tumor cell biology [such as proliferation (Richards et al., 2017), metastasis (Costa -Silva et al., 2015; Soung et al., 2016), drug resistance (Yousafzai et al., 2018), and broad biological processes associated with immunity (Han et al., 2019)], with microRNA (miRNA) playing a significant role. The HCC-derived exosomes have been shown to reduce the cytotoxicity of T cells and NK cells, which are important mediators of the host antitumor immune response and tumor cell immune escape (Chen et al., 2018). Exosomes from highly metastatic MHCC97H cells are connected with low metastatic HCC cells, boosting their migration, chemotaxis, and invasion (Zhang et al., 2020). Exosomal circUHRF1 induces NK cell dysfunction, which causes immunosuppression and may lead to resistance to anti-PD1 therapy in HCC (Greening et al., 2015). Exosomes have been studied for their predictive significance in HCC. However, the true role and mechanism remain unknown. According to a recent study, *BAK1* expression is driven by exosome circ-0051443 *via* competitive interaction with miR-331-3p and acts as a predictor of HCC and a possible therapeutic target (Chen et al., 2020a). Similarly, Huang et al. (2020) discovered that exosomes circRNA-100338 influence cell proliferation, angiogenesis, permeability, angiogenesis mimics, and tumor metastasis. Furthermore, Lu et al. (2020) discovered three lncRNA compositions associated with AFP, implying that they serve as a fingerprint in predicting HCC metastasis. There is little doubt that exosome research provides a novel viewpoint on tumor mechanisms; nevertheless, no systematic examination of exosome biomarkers in HCC patients is reported. The co-expression and competing endogenous RNA (ceRNA) networks may hamper immunotherapy efficacy in HCC *via* PD-L1+ exosome activity (Wei et al., 2021). Furthermore, the lncRNA FAL1 was overexpressed in serum exosomes and acted as a ceRNA pathway to promote cell proliferation and metastasis in HCC (Li et al., 2018a). Moreover, exosomal SENP3-EIF4A1 was reported with the ability to suppress tumor growth *in vivo via* the ceRNA pathway (Wang et al., 2020). However, little attention has been paid to the involvement of exosomes and ceRNA in HCC. Since high-throughput expression data are now available, it is feasible to use global gene expression data to investigate the relationship between exosome analysis and clinical outcomes in HCC patients. The study aimed to explore exosome data from exoRBase database and a free online database to estimate possible binding miRNA from mRNA, lncRNA, and circRNA and discover useful exosome biomarkers for HCC therapy.

## Methods

### Data Collection

The exoRBase (http://www.exoRBase.org) (Li et al., 2018b) is an online database containing circular RNA (circRNA), long noncoding RNA (lncRNA), and messenger RNA (mRNA) extracted from RNA-seq investigations of human blood exosomes. ExoRBase’s initial version comprises 58,330 circular RNAs, 15,501 long noncoding RNAs, and 18,333 mRNAs. The exoRBase also contains 32 normal and 21 HCC samples from which differential mRNA, lncRNA, and circRNA expression between normal and HCC was detected.

### Prediction of the Potential Binding miRNA

By evaluating the expression profiles of the 32 cancer types included in the TCGA study, the ENCORI pan-cancer analysis platform seeks to decode the pan-cancer network of lncRNA, miRNA, pseudogenes, snoRNA, RNA binding protein (RBP), and all protein-coding genes. TargetScan identifies miRNA biological targets by searching for conserved 8mer, 7mer, and 6mer sequences that match the seed region of each miRNA (Lewis et al., 2005). The miRanda is the first miRNA target gene prediction software developed and is not species-specific (Liu et al., 2019), while the miRcode is based on an exhaustive transcriptome prediction of human microRNA targets, including 10,000 long noncoding RNA genes (Jeggari et al., 2012). In addition, the StarBase was designed to decode precancerous and interacting lncRNA, miRNA, competitive endogenous RNA (ceRNA), RBP, and mRNA networks from large-scale CLIP-Seq data and tumor samples (Li et al., 2014). Thus, TargetScan and miRanda databases were used in this study in conjunction with the ENCORI platform to predict possible miRNA binding to mRNA, with only those satisfying both databases being deemed prospective miRNA. The miRcode was used to estimate the potential binding miRNA of lncRNA, and StarBase was utilized to predict the potential binding miRNA of circRNA *via* the ENCORI platform.

### Construction of ceRNA Network

Cytoscape (version 3.7.1) is a bioinformatics software used for constructing and visualizing networks of molecular interactions (Shannon et al., 2003). Cytoscape was also used to create ceRNA networks by constricting the link between mRNAs, lncRNAs, and circRNAs. Furthermore, the DAVID online database (Huang et al., 2009) was also accessed to investigate the network’s mRNAs’ functional annotations and signaling pathways. The Benjamini–Hochberg method was used for converting *p*-values to FDR values. The R (version 3.5.3) and R Bioconductor software packages analyzed all data, while Perl was used for creating data matrixes and performing all the data processing on *p* < 0.05.

## Results

### Differential Expression of RNAs

**FIGURE 1:**
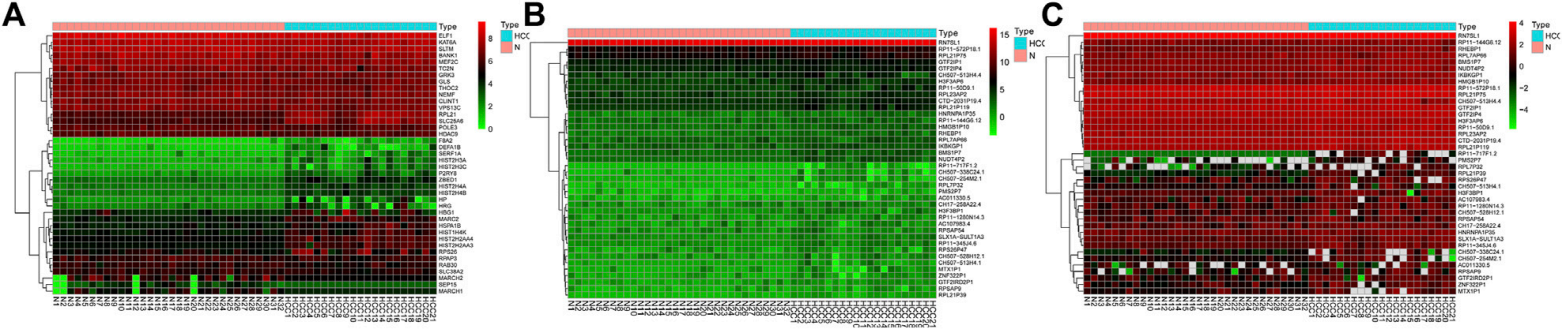
The heatmap of top 20 upregulated and downregulated mRNAs, lncRNAs, and circRNAs. **(A)** mRNAs; **(B)** lncRNAs; **(C)** circRNAs.

After standardizing the microarray results, primarily, the mRNAs, lncRNAs, and circRNAs were selected, which are differentially expressed between normal and HCC samples. The results indicated that a total of 159 mRNAs, 60 lncRNAs, and 13 circRNAs were differentially expressed, with *HIST2H3C* exhibiting the highest log_2_FC change and CTD-2031P19 exhibiting the most relevant lncRNA. Supplementary Tables S1–S3 represented that the most significant circRNA was hsa circ 0001380. Meanwhile, Figure 1 depicts the heatmap of the top 20 upregulated and downregulated mRNAs, lncRNAs, and circRNAs based on the log_2_FC value.

### Prediction of the Potential Binding miRNA and Constrict ceRNA Network

**FIGURE 2:**
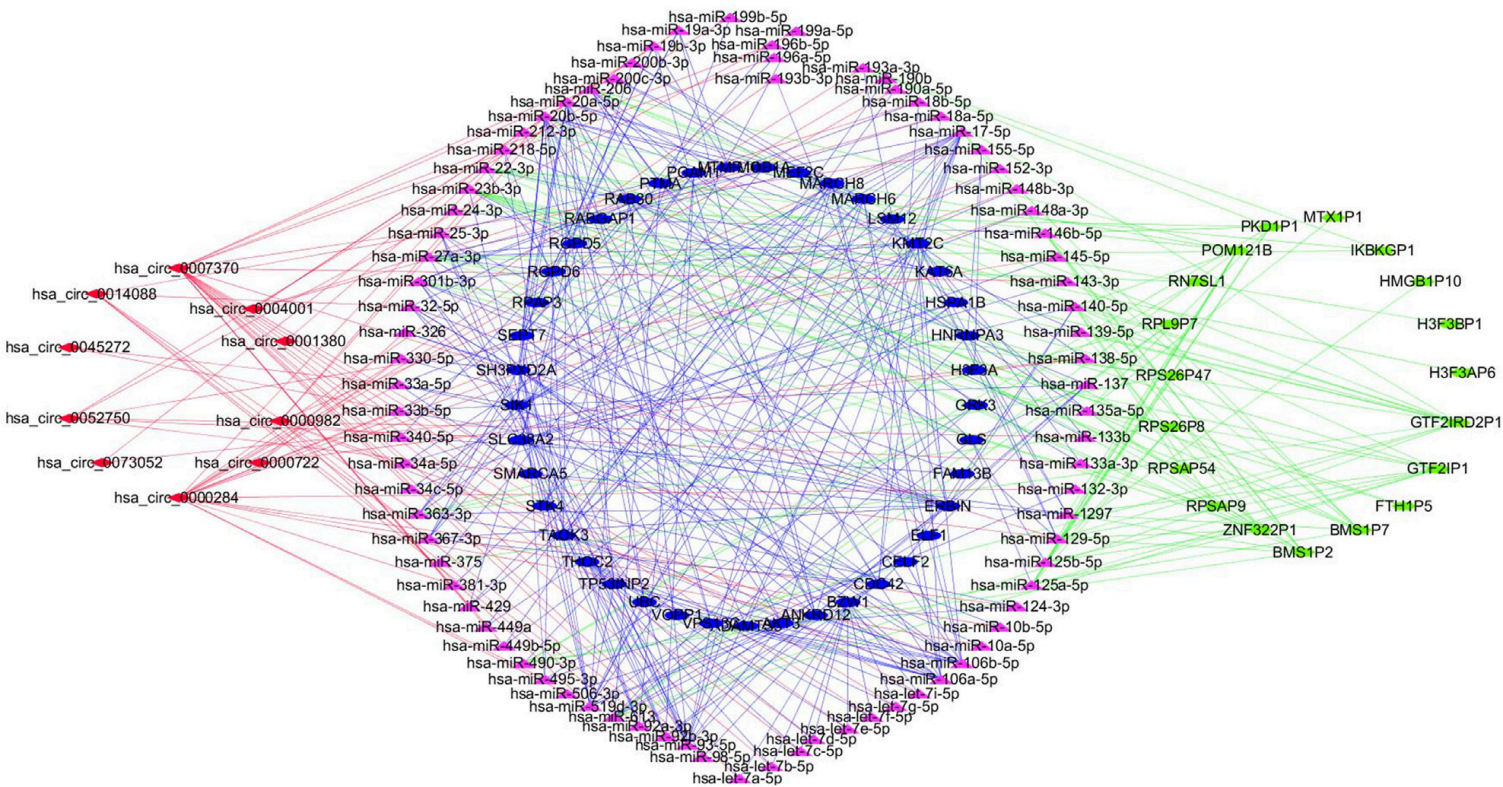
Detailed ceRNA networks and the most significant module were illustrated using Cytoscape.

**FIGURE 3:**
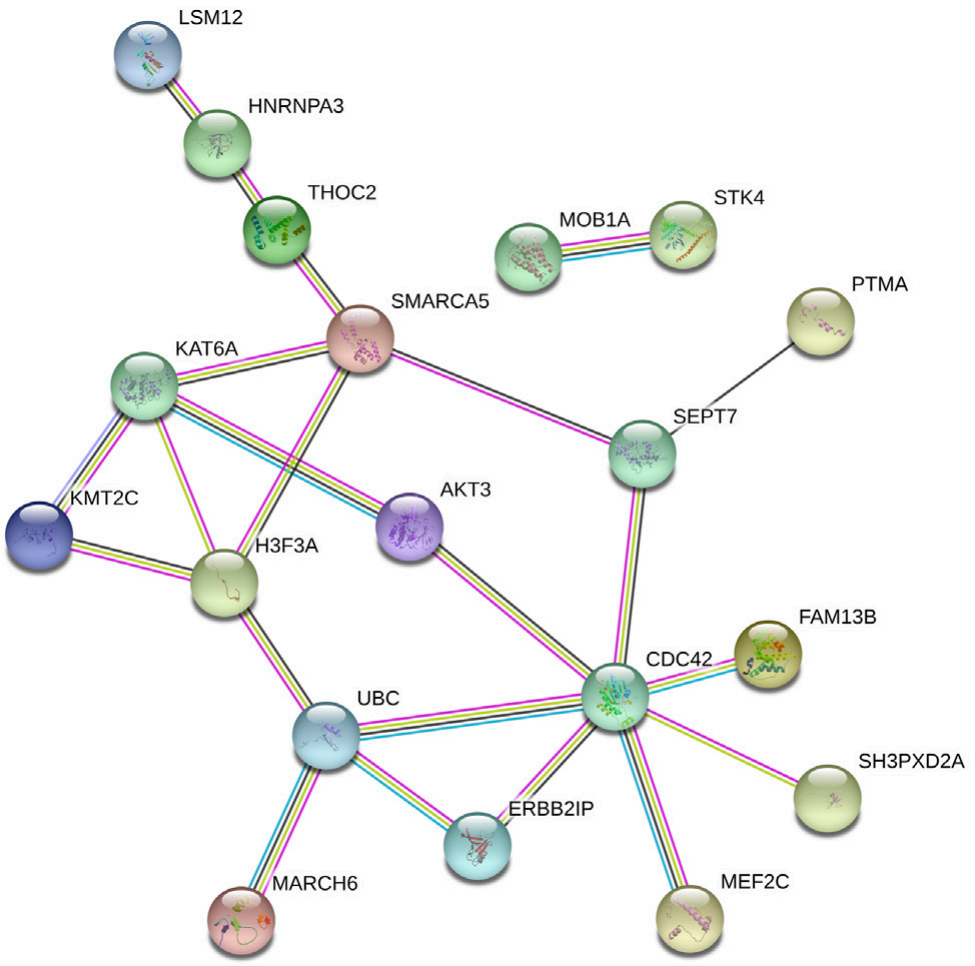
Protein–protein network extracted the mRNA in the ceRNA network.

**FIGURE 4:**
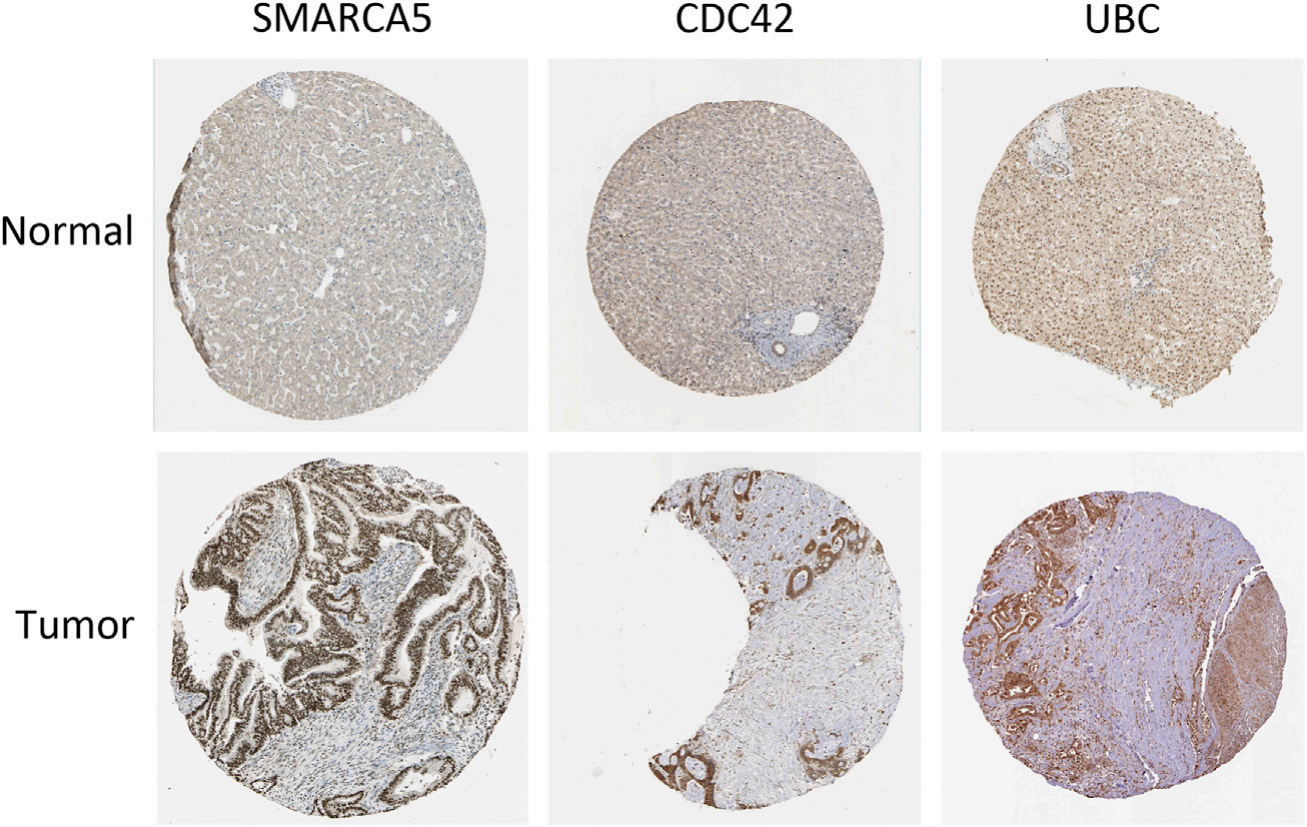
Human Protein Atlas (HPA) online tool directly compares the protein expression of these three genes among normal and cancer tissues.

**FIGURE 5:**
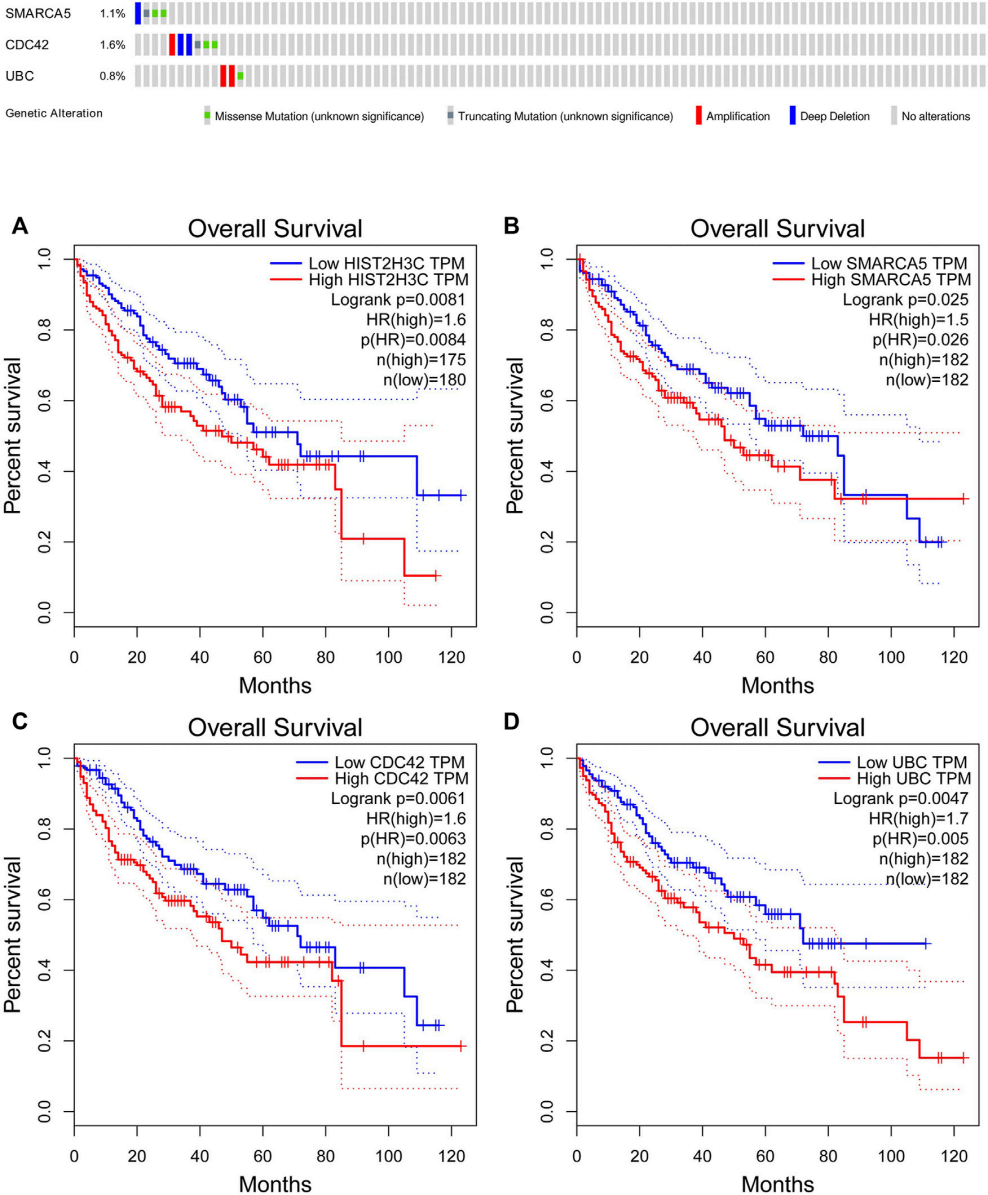
cBioPortal genetic alterations and GEPIA overall survival results. **(A)** HIST2H3C; **(B)** SMARCA5; **(C)** CDC42; **(D)** UBC.

The online database tools were used to determine the possible binding miRNAs of mRNAs, lncRNAs, and circRNAs between the normal and HCC samples. Supplementary Tables S4–S6 contain specific information on each putative miRNA binding partner. Cytoscape was then utilized to create ceRNA networks by constricting the links between mRNAs, lncRNAs, and circRNAs. Cytoscape plug-in Molecular Complex Detection (MCODE) was used to identify densely linked regions in ceRNA networks. Cytoscape enabled the construction of the ceRNA networks, while MCODE helped to select the most significant module within the ceRNA networks. Finally, *MARCH8*, *SH3PXD2A*, has-circ-0014088, hsa-miR-186-5p, and hsa-miR-613 were identified as hub biomarkers with degrees ≥10. The OS analysis revealed no statistically significant difference in survival for *MARCH8* and *SH3PXD2A*, where a high expression of *HIST2H3C* indicated poor survival (Figure 5A). Figure 2 depicts the detailed ceRNA networks and the most significant module. Meanwhile, the mRNA from the ceRNA network was extracted for the purpose of building a protein–protein network, identifying *SMARCA5*, *CDC42*, and *UBC* as the hub genes in the network (Figure 3). Furthermore, immunohistochemical images from the Human Protein Atlas (HPA) online tool were used to directly evaluate the protein expression of these three genes between normal and cancer tissues, and the results showed that the *SMARCA5*, *CDC42*, and *UBC* expression were significantly higher in tumor tissues (Figure 4). Using cBioPortal to investigate genetic modifications in *SMARCA5*, *CDC42*, and *UBC*, it has been reported that the genes were altered in 13 (4%) of 366 HCC patients (Figure 5). The overall survival analysis of *SMARCA5*, *CDC42*, and *UBC* in HCC was also investigated using the Gene Expression Profiling Interactive Analysis (GEPIA) survival analysis module. The statistical difference between the curves was assessed using the log-rank test (Li et al., 2021). The findings revealed that high levels of *SMARCA5*, *CDC42*, and *UBC* expression are associated with poor survival (*p* < 0.05) (Figures 5B–D). The genes discovered in this study may have a role in HCC carcinogenesis.

### GO Enrichment and KEGG Analyses

The DAVID online database was used to investigate the network’s mRNAs’ functional annotation information and signaling pathways. The results of the GO analysis revealed that changes in biological processes (BP) of differentially expressed proteins were enriched in protein phosphorylation, osteoblast differentiation, positive transcription regulation, DNA-templated, and nucleosome assembly. Histone binding and protein kinase activity were the most affected by changes in molecular function (MF). The most abundant cell components (CC) were the cytoplasm, nucleoplasm, and cytosol. According to the KEGG pathway analysis results, the differentially expressed proteins were primarily enriched in the MAPK signaling network, central carbon metabolism in cancer, the glucagon signaling pathway, glutamatergic synapse, and spliceosome (Supplementary Table S7).

## Discussion

Tumor-related exosomes or tumor-derived exosomes are considered critical for controlling carcinogenesis and its development. Identifying and analyzing tumor exosomes can help with early diagnosis, efficacy evaluation, and prognosis analysis of cancers, which are envisaged to significantly advance the oncology research, cancer clinical diagnosis, and introduce novel opportunities for cancer treatment. Some novel and effective exosome biomarkers were identified in this study based on online databases, indicating that the signature has great predictive value. The findings of this study may also represent the exosome status of individuals with liver cancer and prospective biomarkers and therapeutic targets in exosome signaling pathways.

This study used a complete exosome analysis using the exoRBase database on normal and HCC samples; 159 differentially expressed mRNAs, 60 differentially expressed lncRNAs, and 13 differentially expressed circRNAs were identified. The most meaningful mRNAs, lncRNAs, and circRNAs were *RGPD6*, CTD-2031P19.4, and has-circ-0001380, respectively. Microduplication of *RGPD6* was earlier reported in a family with liver cirrhosis and other diseases (Chen et al., 2017). Targets of hsa-miR-363-5p were found in *RGPD6*, and reduced expression of hsa-miR-363-5p was linked to improved overall survival in HCC patients (Zhang et al., 2017). There was no systematic examination of the involvement of *RGPD6*, CTD-2031P19.4, and has-circ-0001380 in HCC yet; hence, the findings of this study may be useful for future research. The ceRNA networks were then built, and *MARCH8*, *SH3PXD2A*, has-circ-0014088, hsa-miR-186-5p, and hsa-miR-613 were identified as hub biomarkers. Membrane-related RING-CH 8 (*MARCH8*) is one of the 11 members of the *MARCH* family of *RING*-finger E3 ubiquitin ligase. Research reports claim that *MARCH8* is an effective antiviral protein that targets viral envelope glycoproteins and reduces their incorporation into the viral particles (Tada et al., 2015). The immunomodulatory role of *MARCH8* in the regulation of intestinal epithelial cells (IEC), MHC II expression, and graft-versus-host disease severity was confirmed (Lineburg et al., 2018). According to a study, the lncRNA *SH3PXD2A*-AS1 knockdown decreased colorectal cancer cell proliferation, migration, and invasion *in vitro* and suppressed carcinogenesis *in vivo* (Ma et al., 2018). *SH3PXD2A*, a gene encoding Tks5, plays a crucial role in invasion, body formation and function, cell migration, and matrix destruction (Pilar et al., 2014). The SH3PXD2A-AS1/miR-330-5p/UBA2 network may influence colorectal cancer growth *via* the Wnt/-catenin pathway (Guo et al., 2021). According to another research, miR-186-5p levels in hepatocellular carcinoma tissues were significantly lower than those in surrounding normal tissues (Lan et al., 2017). The HOXD-AS1/miR-186-5p/PIK3R3 is a novel route in epithelial ovarian cancer that promotes cell migration, invasion, and epithelial-mesenchymal transition (EMT) (Dong et al., 2019); hence, circ-PRKCI may also boost the survival, invasion, and migration of HCC cells by sponging miR-186-5p to increase FOXK1 expression levels (Chen et al., 2021). The miR-613 ectopic expression decreased the proliferation and invasion of Hep3B and SMMC-7721 HCC cells (Wang et al., 2016), whereas the lower miR-613 expression was linked to tumor growth, vascular invasion, and a worse prognosis in HCC patients (Jiang et al., 2018). The RMRP regulates HCC carcinogenesis by functioning as a ceRNA for miR-613 and decreases the miR-613 expression, which is adversely linked with NEAT1 expression in the HCC tissues (Wang et al., 2017; Zhou et al., 2019). There have previously been no similar studies investigating the role of circ-0014088 in malignancies. Therefore, this study may provide potentially useful information for further research. HIST2H3C is a histone H3 gene, and the K27M mutation in HIST2H3C has been reported to cause higher metastatic recurrences in glioma (Castel et al., 2015). The histone H3 variations can control gene expression by altering chromatin organization during cellular processes, contributing to cancer pathogenesis (Rashid et al., 2021). This study discovered some promising prospective exosome indicators in HCC, but further investigation is needed.

The functional annotation information and signaling pathways of the mRNAs in the network were then investigated. According to the KEGG pathway analysis results, the differentially expressed mRNAs were primarily abundant in the MAPK signaling pathway, central carbon metabolism in cancer, the glucagon signaling pathway, glutamatergic synapse, and spliceosome. The Ras/MAPK pathway is activated in 50–100% of human HCC; thus, it is linked to a poor prognosis (Delire and Stärkel, 2015). miR-370 has also been shown in studies to downregulate *BEX2* gene and suppress the activation of the MAPK/JNK signaling pathway, thereby preventing the development of HCC (Wang et al., 2019). Similarly, suppressed BMP2 in HepG2 cells inhibits tumor development, progression, and angiogenesis in HCC *via* inactivating the MAPK/p38 signaling pathway (Feng et al., 2019). As a result, it is anticipated that the differentially expressed mRNAs identified in this study may play an essential role in the MAPK signaling pathway, but further research is required. The TCA cycle is required to handle glucose and glutamine-derived carbon in biosynthetic pathways, which are crucial to tumor growth. According to research, the central carbon metabolic pathway provides energy and produces biosynthetic precursors to aid T-cell homeostasis, proliferation, and immune function (Chen et al., 2020b). The enzymes involved in central carbon metabolism and energy production are essential mediators of bacterial physiology, persistence, and pathogenicity, making them naturally appealing for drug discovery (Hards et al., 2020). This research discovered that these mRNAs were primarily enriched in metabolism-related pathways, and exosomes may be associated with central carbon metabolism and the respiratory chain of *Mycobacterium tuberculosis*. However, the signatures predict that exosomes must be validated in multiple independent cohorts. Though this study provided more information, however, there were some limitations, where the findings have not been validated in clinical samples due to lack of clinicopathologic data, the function of clinical variables in exosomes not being investigated, and the number of genes investigated being insufficient to generalize the importance of exosome transcript upregulation and downregulation in HCC.

## Conclusion

This study discovered atypical signature exosomes for HCC prognostic prediction based on an online database. The signals could mimic exosome microenvironmental disorders providing potential biomarkers for exosome treatment. Future research work is certain to undertake large-scale related research, which will eventually be translated into developing new methods for accurate cancer medicine.

## Data Availability

The original contributions presented in the study are included in the article/Supplementary Material, further inquiries can be directed to the corresponding author.

## Supplementary Material

Supplementary Table S1 | 159 differential mRNA expression and the most meaningful mRNA was *RGPD6*

Supplementary Table S2 | 60 differential lncRNA expression and the most meaningful lncRNA was CTD-2031P19.4.

Supplementary Table S3 | 13 differential circRNA expression and the most meaningful circRNA were has-circ-0001380.

Supplementary Table S4 | Detailed information of mRNA potential binding miRNAs.

Supplementary Table S5 | Detailed information of lncRNA potential binding miRNAs.

Supplementary Table S6 | Detailed information of circRNA potential binding miRNAs.

Supplementary Table S7 | Results of the DAVID online database explored the functional annotation information and signaling pathways of mRNAs.

## Abbreviations

HCC, hepatocellular carcinoma; AFP, alpha-fetoprotein; miRNA, microRNA; ceRNA, endogenous RNA.
